# Comment on “The Vasopressin Loading for Refractory septic shock (VALOR) study: a prospective observational study”

**DOI:** 10.1186/s13054-023-04604-5

**Published:** 2023-08-24

**Authors:** Yuki Kotani, Yoshiro Hayashi

**Affiliations:** 1 Department of Intensive Care Medicine, Kameda Medical Center, 929 Higashi-Cho, Kamogawa, Chiba 296-8602 Japan; 2 Department of Anesthesia and Intensive Care, IRCCS San Raffaele Scientific Institute, Via Olgettina 60, 20132 Milan, Italy; 3 School of Medicine, Vita-Salute San Raffaele University, Via Olgettina 58, 20132 Milan, Italy

Dear Editor,

We read with interest the recent study by Nakamura et al., which investigated the potential use of a 1 U vasopressin bolus to indicate responses to continuous vasopressin infusion in septic shock patients [1]. The authors’ approach of segmenting patients into two groups based on a > 22 mmHg mean arterial pressure (MAP) change unveiled a considerable variation in the decrease of the catecholamine index. The team utilized the MAP response to vasopressin loading to forecast the catecholamine index reduction, a significant addition to our knowledge of vasopressor use.

In this multifaceted field, consensus is gradually forming around the benefits of a personalized multimodal strategy to maximize vasopressor use and maintain the MAP target [2]. Early evidence is amassing to validate this multimodal management [3, 4]. Nakamura et al.’s research aligns with this trend, and their innovative vasopressin administration technique is noteworthy. However, we propose several critical considerations.

First, the researchers permitted individual physicians to set the MAP target, providing MAP values only before and after vasopressin loading. This gap in the data raises questions, considering the primary outcome—the catecholamine index change six hours after vasopressin loading—is considerably influenced by MAP targets. The unpredictable fluctuations in blood pressure following vasopressin loading exacerbate these concerns. Therefore, providing specific data about MAP values six hours after loading would be beneficial.

Second, several key hemodynamic metrics, including the serum lactate level and norepinephrine dose, suggested that the responders were in a less severe stage of septic shock before vasopressin loading. While the study shows MAP changes post-vasopressin loading reliably predicted catecholamine index changes at six hours, such findings should be interpreted cautiously. Specifically, consideration should be given to the added predictive value of a holistic evaluation of hemodynamic status, which remains the gold standard.

Overall, Nakamura et al.’s research significantly contributes to the vasopressor therapy field. However, further studies are necessary to validate the findings and address the critical considerations raised.

## Data Availability

Not available.
